# A novel technique for repositioning a nasobiliary catheter from the mouth to nostril in endoscopic retrograde cholangiopancreatography

**DOI:** 10.1186/s12876-019-1148-0

**Published:** 2019-12-21

**Authors:** Seong Ji Choi, Jae Min Lee, Hyuk Soon Choi, Eun Sun Kim, Bora Keum, Yeon Seok Seo, Yoon Tae Jeen, Hong Sik Lee, Hoon Jai Chun, Soon Ho Um, Chang Duck Kim, Chi Hyuk Oh

**Affiliations:** 1 Division of Gastroenterology and Hepatology, Department of Internal Medicine, Korea University College of Medicine, Seoul, Republic of Korea; 2 Division of Gastroenterology and Hepatology, Department of Internal Medicine, Kyung Hee University College of Medicine, Seoul, Republic of Korea

**Keywords:** Endoscopic retrograde cholangiopancreatography, Endoscopic nasobiliary drainage, Technique

## Abstract

**Background:**

Endoscopic nasobiliary drainage (ENBD) is widely used for biliary decompression in patients with biliary disease. However, it is difficult to reposition a nasobiliary catheter from the mouth to nostril. We developed a new device, which has a curved flexible loop and bar-handle, for repositioning of ENBD catheter. The aim of this study was to evaluate the usefulness of the new loop-device for facilitating the repositioning of an ENBD catheter from the mouth to nostril.

**Methods:**

Between January 2015 and December 2017, a comparative observational study was performed to evaluate the time taken for repositioning a nasobiliary catheter during endoscopic retrograde cholangiopancreatography (ERCP) and compare the results of ENBD procedure between the new loop-device and conventional techniques. In the subgroup analysis, we evaluated the occurrence of oral cavity injury and the time taken to transfer ENBD catheter from the mouth to nostril.

**Results:**

In all, 145 ENBD procedures were performed using these two techniques. The procedure time was significantly shorter in the new technique group than in the conventional group. (44 s vs. 194 s, *p* < 0.001). The total success rate of new device technique was 97.3%. No complication, including oral cavity injury, was observed.

**Conclusions:**

The technique using our new loop-device was useful for repositioning a nasobiliary catheter from the mouth to nostril in ERCP. The new device does not require the removal of the mouthpiece before ENBD positioning, which can help perform the ENBD procedure rapidly and avoid the finger injury of endoscopists.

## Background

Endoscopic retrograde cholangiopancreatography (ERCP) is an important procedure for the treatment of biliary diseases. In patients with cholangitis or biliary obstruction, endoscopic nasobiliary drainage (ENBD) can be required for effective drainage of bile juice [1, 2]. ENBD shows a better effect for decreasing hyperbilirubinemia and has a lower risk of catheter obstruction than endoscopic retrograde biliary drainage [3]. Also, ENBD procedure is usually needed to obtain bile juice sample for cytological analysis or bacteriological culture.

For completing the ENBD after ERCP, a drainage catheter repositioning from the mouth to nostril has to be performed. The conventional technique involves inserting a nasal plastic tube, catching the tip of the tube in the mouth, pulling out the tip, connecting the ENBD catheter with the tube and completing the repositioning by extracting the catheter toward the nose. However, the conventional technique has several disadvantages. The mouthpiece had to be removed from the mouth in sedated patients before the ENBD repositioning. It may take a long time to wait for the patients to wake up. Moreover, the finger injury of the operator often occurs from biting by poorly cooperative patients. When using the forceps, there is a risk of oral cavity injury while grasping the tip of the plastic tube.

Prior investigators have suggested modified techniques to facilitate the ENBD repositioning [4–7]. In a previous study, Hamano et al. suggested a new technique by making a wire loop by crossing guidewire [5]. However, in practice, the crossed guide-wire frequently slips out of the hands and an appropriate loop size or shape has not been defined. Moreover, additional fluoroscopic or endoscopic observation was required for the prior techniques. Poor visualization of the oropharynx inhibits repositioning of the ENBD, especially in patients with a high Mallampati score. Therefore, the shape of the oropharynx should be considered to develop new techniques for the ENBD repositioning.

We have developed a new device composed of a curved wire-loop and bar-handle. In this study, we compared the outcomes between the conventional technique and our new technique using the device we developed for the ENBD repositioning.

## Methods

### Study design and patients

This is a comparative observational study using endoscopic data in patients who underwent ERCP at a tertiary medical center in Seoul, Korea between January 2015 and December 2017. We selected patients who underwent the ENBD procedure with the conventional catheter repositioning technique or the new technique using our device. The inclusion criteria were as follows: (1) age above 18 years, (2) patients who underwent the ENBD procedure with medical records of the procedure time, (3) no medical history of nasopharyngeal deformity, and (4) written informed consent. The conventional catheter repositioning technique was used from January 2015 to August 2016, and the new technique was used since September 2016 in our center. The patients who underwent ERCP from January 2015 to August 2016 were allocated to the conventional technique group, and those who underwent ERCP from September 2016 to December 2017 were allocated to the J-Loop technique group. Total of 160 patients were enrolled, 80 patients for each group, and 9 patients from conventional technique group and 6 patients from J-loop technique group, who were without sufficient medical data such as procedure time record or Mallampati score, were excluded.

This study was conducted in accordance with the Declaration of Helsinki and was approved by the institutional human research committee. The study was reviewed and approved by the Ethics Committee of Korea University Anam Hospital(2019AN0313). All patients signed a written informed consent before the procedure.

### Procedures and device

The conventional technique for ENBD has the following steps: (1) the ENBD catheter located in the oral cavity with the scope is withdrawn from the mouth, (2) the mouth piece is removed and the mouth is opened using the operator’s fingers, (3) a plastic tube is inserted into the nasal cavity, (4) the tip of plastic tube is grasped using fingers or forceps in the oral cavity, (5) the plastic tube is pulled from the oval cavity and connected with the ENBD catheter, and (6) the plastic tube, connected with the ENBD catheter, is pulled toward the nose.

**Fig. 1:**
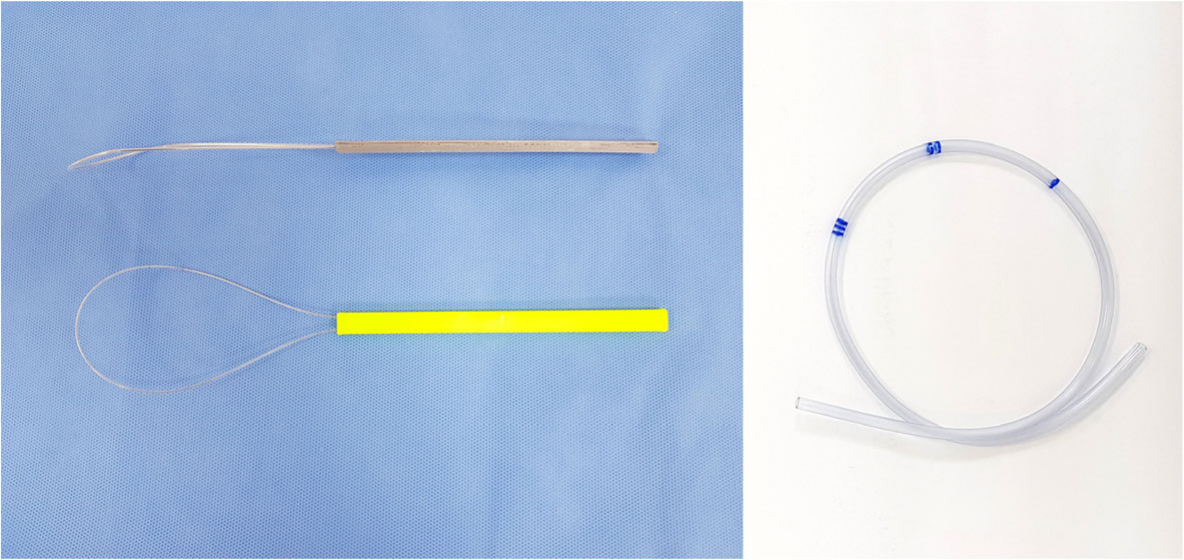
New device: J-loop and plastic tube with marking

**Fig. 2:**
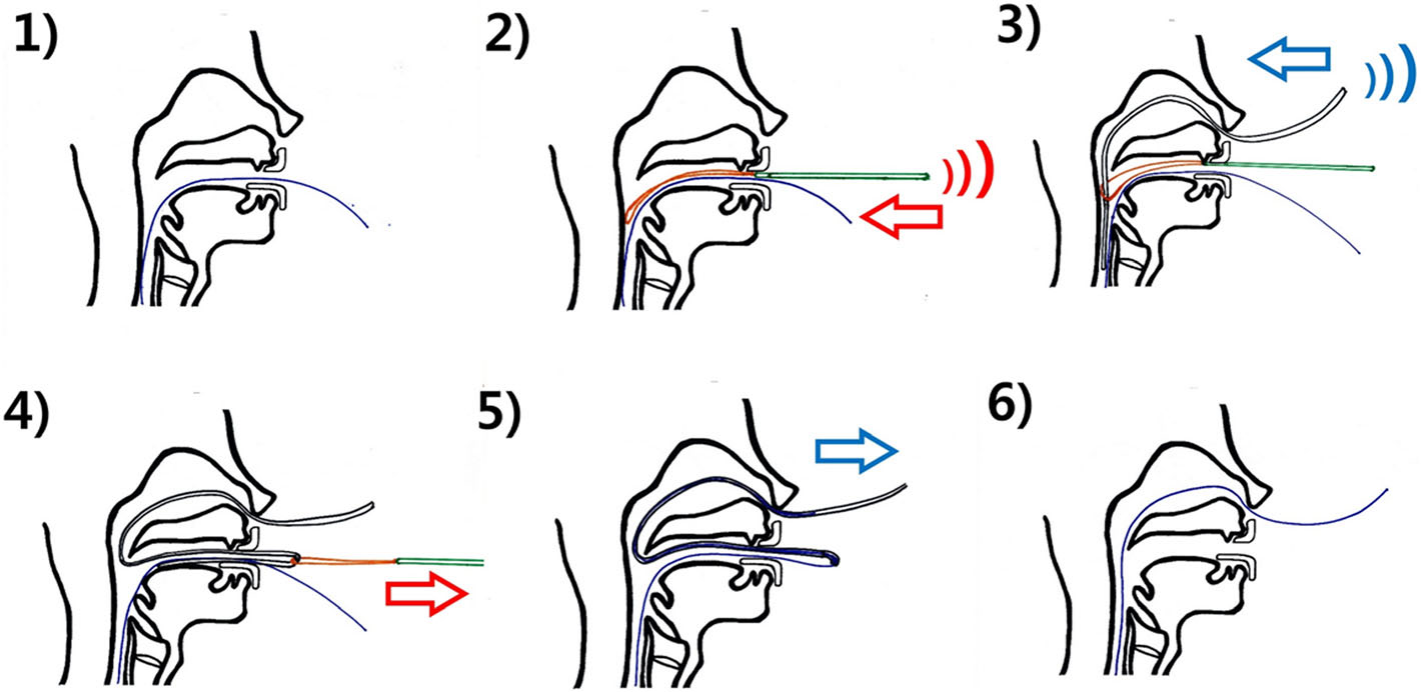
Procedure with the new technique using J-loop for repositioning of the ENBD catheter


The new technique was performed by using the new device. The device set composed of a loop-shaped holder and a plastic tube with markings (Fig. 1, patent pending). The loop-shaped holder named as ‘J-Loop’ consisted of a curved loop head and a bar-shaped handle. The plastic tube has several markings for measuring the insertion depth. The new technique has the following steps: (1) the ENBD catheter located in oral cavity with the scope is withdrawn from the mouth, (2) the J-loop is inserted into the oral cavity, (3) the plastic tube is inserted into the into the nasal cavity up to markings, (4) the plastic tube, caught in the loop, is pulled, and (5) the plastic tube, connected with the ENBD catheter, is pulled toward the nose. Figure 2 and Additional file 1: Video S1 show each step of the new technique using J-Loop. The ENBD catheter and the J-loop can be visualized under fluoroscopy (Additional file 2: Figure S1).

**Additional file 1: Video S1.** Procedural video of nasobiliary catheter reposition using J-loop


### Outcomes of procedures

The results of the conventional technique and the new technique using J-Loop were compared. The outcomes were ENBD catheter positioning time, success rate and side effects. The ENBD positioning time was defined as the time from ‘scope withdrawn from the mouth’ to ‘completion of fixation of ENBD catheter toward the nose.’ The shape of the oropharynx was scored by using the Mallampati score [7].

### Statistical analysis

Normally distributed data were expressed as mean ± standard deviation (SD), and student t-test was used to compare the parameters. Not normally distributed data were expressed as median (range), and Mann-Whitney U test was used to compare the parameters. Chi-square test or Fisher’s exact test was used for compare the distribution of a categorical variable. *P* values < 0.05 were considered statistically significant. Data were analyzed using the Statistical Package for the Social Sciences version 24.0 (IBM Corp., Armonk, N.Y., USA).

## Results

A total of 145 patients were included in this study. The baseline characteristics of the patients are shown in Table 1. The mean age was 65.7 ± 14.7 years, and 75 (52%) patients were men. The conventional technique and the new technique using J-Loop were performed in 71 and 74 patients, respectively, for the ENBD repositioning. Table 2 shows the outcomes of the two techniques for the ENBD repositioning in ERCP. The new technique using J-Loop showed significantly shorter procedure time for the ENBD repositioning than the conventional technique (44 s vs. 194 s, *P* < 0.001). The technical success of the new technique was achieved in 97.3% (72/74) of cases. Two patients who underwent the new technique experienced difficulty due to deep insertion of the J-loop into the throat, and were barely successful with conventional method. A high Mallampati score was related to difficult ENBD repositioning using the conventional technique. However, there was no significant difference in the repositioning time using J-Loop (Table 3). In the new technique group, there were no complications such as oral cavity injury, tooth damage, and pharynx bleeding, nor any unexpected event, such as an injury to the operator’s finger.

**Table 1:** Baseline characteristics of the enrolled patients underwent ERCP

	All patients (*n* = 145)	Conventional technique group (*n* = 71)	J-Loop technique group (*n* = 74)	*P-value*
Age, years	65.7 ± 14.7	67.7 ± 13.4	63.9 ± 15.8	0.124
Male, *n* (%)	75 (51.7)	38 (53.5)	37 (50.0)	0.671
Indication for ERCP, *n* (%)				0.154
Gallstone disease	88 (60.7)	46 (64.8)	42 (56.8)	
Malignant bile duct obstruction	23 (15.9)	13 (18.3)	10 (13.5)	
Benign bile duct stricture	25 (17.2)	11 (15.5)	14 (18.9)	
Bile leak	4 (2.8)	0 (0)	4 (5.4)	
Others	5 (3.4)	1 (1.4)	4 (5.4)	
Mallampati score, *n* (%)				0.686
Class 1	82 (56.6)	42 (59.2)	40 (54.1)	
Class 2	33 (22.8)	15 (21.1)	18 (24.3)	
Class 3	18 (12.4)	7 (9.9)	11 (14.9)	
Class 4	12 (8.3)	7 (9.9)	5 (6.8)	

*ERCP* endoscopic retrograde cholangiopancreatography

**Table 2:** Clinical outcomes of ENBD procedure

	Conventional technique group	J-Loop technique group	*P-value*
Patients, *n*	71	74	
ENBD repositioning time, s (range)	194.0 (88–544)	34.5 (19–150)	< 0.001
Technical success, *n* (%)	71 (100)	72 (97.3)	0.163

*ENBD* endoscopic nasobiliary drainage

*s* second

**Table 3:** Mallampati score and ENBD repositioning time

	Low Mallampati score (1 or 2)	High Mallampati score (3 or 4)	*P-value*
Patients, *n*	115	28	
ENBD repositioning time, s (range)			
Conventional technique	171.2 (88–264)	286.2 (205–544)	< 0.001
J-Loop technique	42.9 (19–146)	49.9 (27–150)	0.482

*ENBD* endoscopic nasobiliary drainage

*s* second

## Discussion

In this study, the new ENBD device, J-Loop, was easy, convenient, fast and safe for repositioning the ENBD catheter from the mouth to nostril. The outcomes showed that the new technique is more efficient than the conventional technique for the ENBD repositioning. The conventional technique took an average of 194 s for the ENBD repositioning, which was similar to the results from other studies [4, 5]. Using the new technique, the time required for the ENBD repositioning was reduced to 44 s (approximately 77%). As the steps of removing the mouthpiece and waiting for the patient to wake up to open their mouth was skipped, the overall procedure to complete ENBD was simplified.

In the conventional technique, operators had to insert a plastic tube into the nose and pull out its tip through the pharynx and oral cavity using a finger or forcep. However, it is difficult to catch the tip of a plastic tube especially in patients with a short neck or a small oral cavity. Poor visualization of the oral cavity is an obstacle to a successful ENBD repositioning. When using the conventional technique, the ENBD repositioning time was significantly longer in the higher Mallampati score patients than that in the lower Mallampati score patients.

However, the new technique does not require observing the oral cavity or catching the tip of a plastic tube. As the plastic tube can be dragged out while pulling out the J-loop, operators just have to push the J-loop into the mouth and plastic tube into the nostril up to a marking, and pull the J-loop out. Appropriate depths were marked on plastic tube. There was no significant difference related to the Mallampati scores of the group while using the new technique.

Another advantage of the new repositioning technique is that the ENBD repositioning with J-loop can be performed to the sedated patient immediately after removing the endoscope. As the operator does not need to touch the patient’s oral cavity, there is no risk of causing injury to the patient, such as a mucosal scratch or broken tooth. Moreover, as the entire procedure is completed in the sedated state, the patient does not feel any discomfort during the ENBD repositioning. Above all, the operator is able to avoid an unexpected injury or trauma to the finger and can be free from the fear of ENBD, either fear of failure or pain. This study was performed in patients who underwent ERCP under sedation, not under general anesthesia with endotracheal intubation. Therefore, further studies are needed to determine whether the J-Loop is also convenient for ERCP under general anesthesia.

## Conclusions

In summary, the new technique is useful and effective for repositioning of the ENBD catheter from the mouth to nostril. The technique using the J-loop allows the operator to easily maneuver the ENBD procedure and helps avoid injury to both the operator and patients.

## Supplementary information

**Additional file 2: Figure S1.** Visualization of nasobiliary catheter under fluoroscopy

## Data Availability

The data of this study are available from the corresponding author upon reasonable request.

## Abbreviations

ENBD: Endoscopic nasobiliary drainage
ERCP: Endoscopic retrograde cholangiopancreatography
SD: Standard deviation
