# Supplementary figures and images for: A novel technique for repositioning a nasobiliary catheter from the mouth to nostril in endoscopic retrograde cholangiopancreatography
Source: BMC Gastroenterol. 2019 Dec 21;19:222. doi: 10.1186/s12876-019-1148-0 (PMC6925839; doi:10.1186/s12876-019-1148-0)

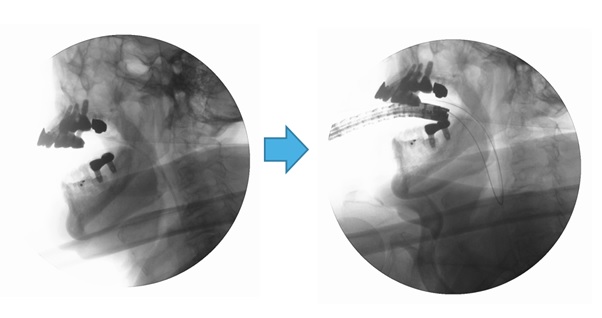

Supplement: Supplementary file 2 — Additional file 2: Figure S1. Visualization of nasobiliary catheter under fluoroscopy [file 12876_2019_1148_MOESM2_ESM.jpg]
